# Predominance of Heart Failure With Preserved Ejection Fraction in Postmenopausal Women: Intra- and Extra-Cardiomyocyte Maladaptive Alterations Scaffolded by Estrogen Deficiency

**DOI:** 10.3389/fcell.2021.685996

**Published:** 2021-09-29

**Authors:** Adebayo Oluwafemi Adekunle, Gabriel Komla Adzika, Richard Mprah, Marie Louise Ndzie Noah, Joseph Adu-Amankwaah, Ruqayya Rizvi, Nazma Akhter, Hong Sun

**Affiliations:** ^1^Department of Physiology, Xuzhou Medical University, Xuzhou, China; ^2^Xuzhou Medical University, Xuzhou, China

**Keywords:** HFpEF, estrogen deficiency, β-adrenergic receptors, calcium handling, cardiomyocyte cytoskeleton, extra cellular matrix, coronary microvascular endothelial, inflammation

## Abstract

Heart failure (HF) remains a public health concern as it is associated with high morbidity and death rates. In particular, heart failure with preserved ejection fraction (HFpEF) represents the dominant (>50%) form of HF and mostly occurring among postmenopausal women. Hence, the initiation and progression of the left ventricular diastolic dysfunctions (LVDD) (a typically clinical manifestation of HFpEF) in postmenopausal women have been attributed to estrogen deficiency and the loss of its residue cardioprotective effects. In this review, from a pathophysiological and immunological standpoint, we discuss the probable multiple pathomechanisms resulting in HFpEF, which are facilitated by estrogen deficiency. The initial discussions recap estrogen and estrogen receptors (ERs) and β-adrenergic receptors (βARs) signaling under physiological/pathological states to facilitate cardiac function/dysfunction, respectively. By reconciling these prior discussions, attempts were made to explain how the loss of estrogen facilitates the disruptions both ERs and βARs-mediated signaling responsible for; the modulation of intra-cardiomyocyte calcium homeostasis, maintenance of cardiomyocyte cytoskeletal and extracellular matrix, the adaptive regulation of coronary microvascular endothelial functions and myocardial inflammatory responses. By scaffolding the disruption of these crucial intra- and extra-cardiomyocyte physiological functions, estrogen deficiency has been demonstrated to cause LVDD and increase the incidence of HFpEF in postmenopausal women. Finally, updates on the advancements in treatment interventions for the prevention of HFpEF were highlighted.

## Introduction

Heart failure (HF) remains a public health concern as it is associated with high rates of morbidity and deaths that ultimately result from cardiovascular diseases (Roger, 2013). Left ventricular ejection fraction (LVEF) is a crucial index in ascertaining the extent of cardiac dysfunction in HF patients. Hence, by the characterization of the state of the left ventricular’s functionality in ejection fraction, HF is classified into three subtypes, namely; Heart failure with reduced ejection fraction (HFrEF) (LVEF ≤ 40%), heart failure with mid-range ejection fraction (HFmEF) (LVEF 40–49%), and heart failure with preserved ejection fraction (HFpEF) (LVEF ≥ 50%) (Masri et al., 2017; Özlek et al., 2019). Both HFrEF and HFmEF are the predominant forms of HF in men, while HFpEF represents the dominant form of HF, mostly among postmenopausal women (Özlek et al., 2019). Even so, HFpEF accounts for nearly 50% of the HF recorded according to clinical demographics (Lam et al., 2011; Ma et al., 2020). The typical clinical presentation of HFpEF is a left ventricular diastolic dysfunction (LVDD) with an intact systolic function along with other HF signs and symptoms such as shortness of breath, fatigue, and chest pain, among others (Ma et al., 2020).

Circulating catecholamines facilitates the heart’s inotropy and chronotropic functions by signaling via β-adrenergic receptors (βARs) under physiological state. However, hyperactivity of the sympathetic nervous system (SNS) during stress upregulates circulating catecholamines, which overstimulates βARs, causing the receptors to become dysfunctional, leading to their desensitization and downregulation (Adzika et al., 2019). Unfortunately, the calcium handling proteins, such as the L-type calcium channel (LTCC), which ensure homeostatic levels of Ca^2+^ in cardiomyocytes for proper cardiac contractions, are downstream of the βARs (Aikawa et al., 2017; Ahmad and Gerson, 2018; Morimoto et al., 2020). Hence, the dysfunctionalities of the receptors during chronic catecholamine stress disrupts the intracellular Ca^2+^ and cardiac contractility. Also implicated in the pathogenesis and exacerbation of HFpEF are the alterations in myocardial extracellular matrix (ECM), cardiac cytoskeletal proteins, and coronary microvascular endothelial (CME) dysfunctionalities, plus maladaptive inflammatory responses in the myocardia. Regardless, estrogen (E2) and estrogen receptors (ERs) have been shown to adaptively modulate the expressions, responses, and activities of the aforementioned factors implicated in the initiation and progression of HFpEF (Sophonsritsuk et al., 2013; Michalson et al., 2018). Hence, a lower incidence of the disease condition is observed in premenopausal women than postmenopausal women and males of all age cohorts (Michalson et al., 2018; Sabbatini and Kararigas, 2020; Ndzie Noah et al., 2021).

This review seeks to discuss the probable mechanisms aggravating the incidence of HFpEF in postmenopausal women from a pathophysiological and immunological standpoint. The initial discussion recaps E2 and ERs and βARs signaling under physiological/pathological states to facilitate cardiac function/dysfunction, respectively. Finally, by reconciling these prior discussions, we explain the possible pathomechanisms of how E2 deficiency scaffolds signaling cascades that hastens the incidence of HFpEF in postmenopausal women and provided updates on the advancements in treatment interventions.

## Physiological and Pathophysiological Interplays Between E2 and ERs, and βARs in Cardiac Function Modulation

### Physiological Interplays

**FIGURE 1:**
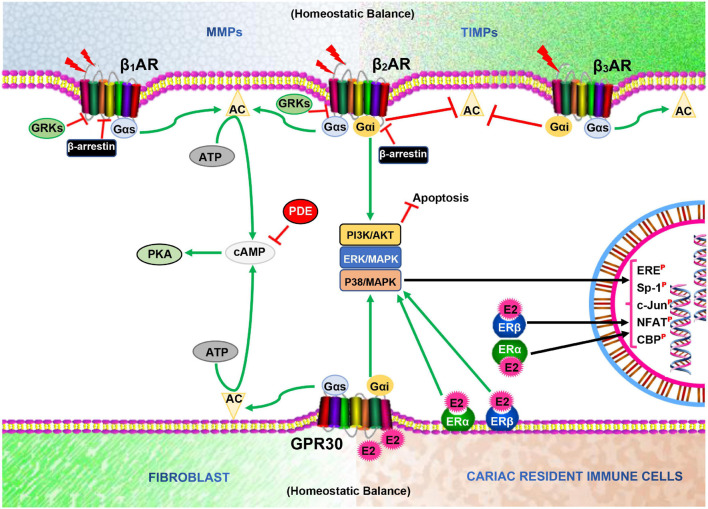
Illustration of homeostatic state between MMPs and TIMPs, and Fibroblast and Cardiac resident immune cells as well as β-adrenergic receptors (βARs) and Estrogen receptors (ERs) signaling interplays in the cardiomyocytes. AC, Adenylyl cyclase; cAMP, Cyclic adenosine monophosphate; PKA, Protein kinase A; GRKs, G protein-coupled receptor kinases; E2, Estrogen; PDE, Phosphodiesterase; ERE, Estrogen receptor element; Sp-1, Stimulating protein-1; NFAT, Nuclear factor of activated T cells; ^
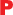
^, Phosphorylated; 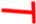, Inhibit; 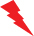, Catecholamine.

ERs (ERα, ERβ, and GPR30) and βARs (mainly, β_1_AR, and β_2_AR) are both predominate seven transmembrane-spanning receptors expressed in cardiomyocytes, cardiac fibroblast, and vascular tissues, and mediates signaling crucial for their cellular functions (Mahmoodzadeh et al., 2010). Typically, ERs mediate estrogenic responses, which are classified as rapid/non-genomic (via GPR30) and non-rapid/genomic (via ERα and ERβ) (Asokan Shibu et al., 2017; Ndzie Noah et al., 2021). Recently, it has been suggested that GPR30 could indirectly induce genomic effects through interactions with ERα and ERβ to facilitate physiological functions (Fuentes and Silveyra, 2019). Meanwhile, under physiological conditions, β_1_AR and β_2_AR mediates catecholamine responses rapidly via G_αs_/cAMP/PKA and/or G_αi_/PI3K/Akt to facilitate cardiac inotropy or prevent cardiac insults (Adzika et al., 2019). Similar to β_2_AR, GPR30 is capable of pleiotropic signaling via G_αs_/cAMP/PKA and/or G_αi_/PI3K/Akt in cardiomyocytes; hence, this is suggestive of their possible multi-interplays in signaling to facilitate proper cardiac functions, as demonstrated (Hou et al., 2018; Machuki et al., 2018; Möller et al., 2018; Ndzie Noah et al., 2021) (Figure 1). In cardiomyocytes, the activation of cAMP/PKA downstream GPR30 and βARs results in the phosphorylation of various calcium handling proteins such as LTCC, ryanodine receptor (RyR), phospholamban (PLB), sarcoplasmic reticulum Ca^2+^ ATPase (SERCA), and sodium-calcium exchanger (NCX), as well as myofilament proteins [cardiac troponin I (cTnI), myosin binding protein-C (MyBP-C and titin)] to facilitate inotropy, chronotropic, and relaxation (Tilley, 2011; Najafi et al., 2016). While this signaling is prototypic of βARs activation by catecholamine under physiological conditions, E2 and ERs have been shown in our previous studies to adaptively modulate the cascade by enhancing βARs expression and activities (Hou et al., 2018; Machuki et al., 2019; Ndzie Noah et al., 2021). Furthermore, the cardiac cytoskeletal proteins (such as titin) and ECM [comprising of matrix metalloproteinases (MMPs) and tissue inhibitors of matrix metalloproteinases (TIMPs)] perform essential functions for maintaining the myocardial architecture. Also, other cellular constituents; namely; fibroblasts and resident immune cells, which make up the myocardial architecture and ensure proper cardiac functions, have been shown to be adaptively modulated by the direct or indirect interactive signaling of βARs and ERs (Kim et al., 2002; Chancey et al., 2005; Rienks et al., 2014; El Hajj et al., 2017; Ambhore et al., 2019).

### Pathophysiological Interplays

Over the recent decades, attempts to elucidate the pathomechanisms expediting the incidence of cardiovascular diseases (including HFpEF) in postmenopausal women have revealed that the synergy of βARs hyperstimulation and E2 deficiency permits maladaptive signaling cascades which drives the adverse cardiac remodeling (Sherwood et al., 2010; Fares et al., 2013; Machi et al., 2016; Hou et al., 2018; Ndzie Noah et al., 2021). Apparently, the loss of E2 during menopause permits an increase in SNS activities, hence, increases the level of circulating catecholamines which overstimulates the βARs and causes their desensitization, depletion, and dysregulation. This implicates βARs (mainly, β_2_AR) in the pathogenesis of LVDD and, ultimately, HFpEF (Brooks and Conrad, 2009; Wang et al., 2019; Wu et al., 2019). Meanwhile, the presence of E2 would have averted the initiations of the cardiac dysfunction via multiple mechanisms such as: (1) Modulating the activities of the SNS to keep the levels of circulating catecholamines normal to ensure proper cardiac health. (2) Mitigating the dysregulation of βARs by regulating G-protein-coupled receptor kinases (GRKs) and other kinases that disrupt the receptor’s physiological functions (Abraham et al., 2018; Adzika et al., 2019). (3) Signaling via GPR30/G_ai_/PI3K/Akt to compliment β_2_AR/_Gai_/PI3K/Akt signaling to reenforce the attenuation of any adverse cardiac remodeling that may distort the myocardial architecture (ECM and cardiac cytoskeletal proteins) and ultimately induce HFpEF (El Hajj et al., 2017; Ndzie Noah et al., 2021). (4) Preventing βARs-induced Ca^2+^ hypersensitivity of the cardiac contractile apparatus, which results in LVDD (Thawornkaiwong et al., 2007; Hamdani et al., 2013). (5) Sustaining the bioavailability of nitric oxide (NO) for CME vasodilatory function via GPR30/G_ai_-induced Akt, ERK1/2, or CaMK-II phosphorylation of endothelial nitric oxide synthase (eNOS) at Ser1177 when β_2_AR/G_ai_/PI3K/Akt-induced NO production is dysregulated, thereby, reduces the initiation or progression toward HFpEF (Fulton et al., 1999; Kolluru et al., 2010; Sabbatini and Kararigas, 2020; Ndzie Noah et al., 2021). (6) Downregulating inflammatory cytokines; interleukin (IL)-6 and tumor necrosis factor-alpha (TNFα) as well as NFκB to prevent excess fibroblast activation and proliferation, which distorts the ECM homeostasis and hastens LVDD/HFpEF progression (McLarty et al., 2013; Ambhore et al., 2019; Sabbatini and Kararigas, 2020). However, unlike premenopausal women, postmenopausal women lose all these multiple cardioprotective mechanisms mediated and exerted by E2 and ERs directly or indirectly via βARs modulations.

### Heart Failure With Preserved Ejection Fraction Pathomechanism Scaffolds Facilitated by Estrogen Deficiency

Herein, the discussions will be centered on explaining the probable pathomechanisms involved in the initiation and progression of HFpEF in postmenopausal women, mostly due to the synergy of E2 deficiency and βARs dysfunctionalities.

### Intra-Cardiomyocyte Maladaptive Alterations

#### Disruption of Ca^2+^ Handling Homeostatic Functions

Focusing on individual calcium handling proteins to assess the extent of their alterations and dysfunctionalities resulting from E2 deficiency reveals multiple vulnerabilities in the Ca^2+^ cycle that might explain the predisposition of postmenopausal women to HFpEF.

**FIGURE 2:**
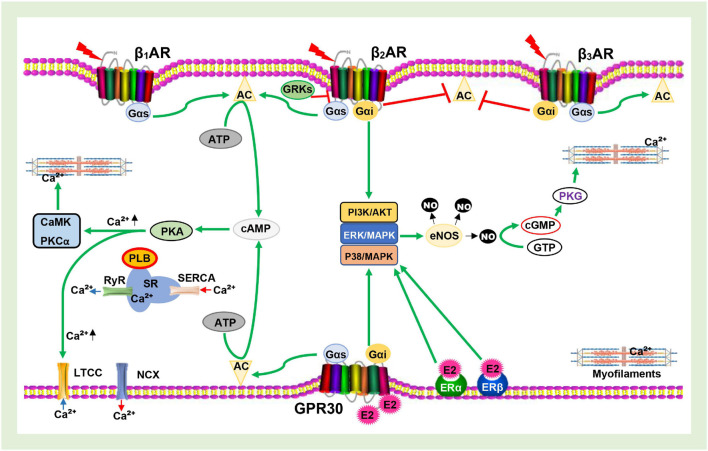
Schematics of Calcium channel cascades and Myofilament activation being facilitated by β-adrenergic receptors (βARs) and Estrogen receptors (ERs signaling. AC, Adenylyl cyclase; cAMP, Cyclic adenosine monophosphate; PKA, Protein kinase A; PKCα, Protein kinase C alpha; GRKs, G protein-coupled receptor kinases; E2, Estrogen; eNOS, Endothelial nitric oxide synthase; NO, Nitric oxide; PDE, Phosphodiesterase; SR, Sarcoplasmic reticulum; PLB, Phospholamban; RyR, Ryanodine receptor; SERCA, Sarcoplasmic reticulum Ca^2+^ ATPase; LTCC, L-type calcium channel; NCX, Sodium-calcium exchanger; CaMK, Calmodulin- dependent kinase; cGMP, Cyclic guanosine monophosphate; GTP, Guanosine triphosphate; PKG, Protein kinase G; ^
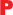
^, Phosphorylated; 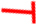, Inhibit; 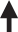, Upregulate; 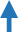, Ca^2+^ entry/release; 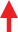, Ca^2+^ uptake/expulsion; 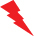, Catecholamine.

In cardiomyocytes, Ca^2+^ regulates both mitochondrial and contractile activities; hence, it plays a crucial role in sustaining the energy demands and supplies of the myocardium. Although primarily regulated by signaling via βARs, E2 and ERs have also been shown to influence the expression of the receptor and its downstream proteins (PKA, LTCC, PLB, SERCA, RyR, and NCX) involved in maintaining the intracellular calcium homeostasis (Michalson et al., 2018; Lai et al., 2019; Machuki et al., 2019; Maslov et al., 2019). As depicted in Figure 2, the initial phase of the PKA-dependent calcium cycle involves LTCC allowing entry of Ca^2+^ into cardiomyocytes upon phosphorylation and the influx of Ca^2+^ into the cytosol from the sarcoplasmic reticulum (SR) via RyR to facilitate contraction. The latter phase, which ensures relaxation, requires that the excess Ca^2+^ be taken back into the SR via SERCA and/or expelled out of the myocyte via NCX (Fearnley et al., 2011; Machuki et al., 2018; Jiao et al., 2020). Hence, the inability to effectively carry out the cascades required for the latter results in LVDD, a typical characteristic of HFpEF (Kilfoil et al., 2020).

In their attempt to investigate the disease mechanism, Kilfoil et al. demonstrated that βARs are mostly desensitized in HFpEF models, therefore, might account for the dysregulation in calcium handling downstream (Kilfoil et al., 2020). Even so, the phosphorylation of cTnI and MyBP-C by PKA does reduce the affinity of troponin C for Ca^2+^ and the acceleration of the cross-bridge detachment to hasten myocardial relaxation (Matsuba et al., 2009). However, in HFpEF, these two proteins are less phosphorylated (Hamdani et al., 2013). E2 being able to prevent βARs desensitization by inhibiting GRKs would have impeded dysfunctionalities of the receptor and enhanced the phosphorylation of cTnI and MyBP-C by PKA. This would have ensured proper calcium handling to circumvent the LVDD as suggested (Maslov et al., 2019); however, E2 deficiency in postmenopausal women leaves them vulnerable.

E2 has been extensively shown to decrease LTCC expression as well as regulate its gating activities to ensure only a sufficient amount of Ca^2+^ enters the cardiomyocyte (Kurata et al., 2001; Chu et al., 2006; Brewer et al., 2009; Sribnick et al., 2009; Harvey, 2012; Lai et al., 2019). Contrarily, ovariectomy (mimicking E2 loss) resulted in the upregulation of the LTCC and increased Ca^2+^ entry (Chu et al., 2006; Brewer et al., 2009; Lai et al., 2019). Also, it has been demonstrated that E2 stabilizes the Ca^2+^ holding and Ca^2+^ gating activities of the SR and RyR, respectively. As such, the deficiency of E2 was observed to induce SR Ca^2+^ overload, increased cardiomyocyte contraction, and the amplitude of Ca^2+^ transient (Parks et al., 2017; Yang et al., 2017; Jiao et al., 2020). In addition, RyR was found to be highly phosphorylated at Ser2808, causing an increased release of Ca^2+^ besides being “leaky” in ovariectomy animal models (Ai et al., 2005; Kravtsov et al., 2007; Györke and Carnes, 2008; Fares et al., 2012; Kilfoil et al., 2020). However, the replacement of E2 reverted these abnormalities in SR and RyR Ca^2+^ handling functions (Kravtsov et al., 2007; Parks et al., 2017; Yang et al., 2017). Although it might be argued that these initial phase cascades (Ca^2+^ entry and Ca^2+^ release) of the Ca^2+^ cycle are responsible for contraction, any inefficiencies occurring here may indirectly impact negatively on the latter phase cascade (Ca^2+^ uptake and Ca^2+^ expulsion), as the amount of Ca^2+^ influx in the cytosol might exceed the Ca^2+^ hold capacity plus Ca^2+^ efflux capability. Consistent with these inferences, Miranda et al. suggested that SR Ca^2+^ release and SR Ca^2+^ uptake are impaired during HFpEF (Miranda-Silva et al., 2020).

Furthermore, Ca^2+^ uptake via SERCA aids partly in restoring intracellular Ca^2+^ levels to ensure the relaxation of cardiomyocytes. Nonetheless, in HFpEF models, the expression and function of SERCA are reportedly downregulated (Miranda-Silva et al., 2020). This phenomenon is consistent with observations in ovariectomized animal models (Bupha-Intr and Wattanapermpool, 2006). Also, in conformity with the finding from ovariectomized animal models, Miranda et al. and others have observed lower SERCA/PLB ratio and decreased phosphorylated PLB in HFpEF models (Dupont et al., 2012; Rouhana et al., 2019; Miranda-Silva et al., 2020). Regardless, E2 and ERs have been widely demonstrated to promote the functions of SERCA by upregulating its expression, maintaining SERCA/PLB ratio, and enhancing PLB phosphorylation to increase Ca^2+^ uptake (Ren et al., 2003; Chu et al., 2006; Turdi et al., 2015; Machuki et al., 2019). In addition, the functionality of NCX is crucial in complimenting SERCA/PLB in restoring intracellular Ca^2+^ levels. Although the effect of E2 on NCX expressions and activities remains inconclusive (Müller-Ehmsen et al., 2001; Kravtsov et al., 2007; Chen et al., 2011; Yang et al., 2017), it is suggested that E2 normalizes its expression and current density to enhance Ca^2+^ expulsion. If not so, NCX might fail to ensure calcium homeostasis either by ineffective expulsion of Ca^2+^ or switch into the reverse mode to increase the accumulation of intracellular calcium along with increased intracellular Na^+^ as reported in HFpEF (Louch et al., 2010). Besides, E2 and ERs have been shown to decrease the sensitivity of myofilaments to Ca^2+^ so as to enhance myocardial relaxation and prevent HFpEF (Fares et al., 2013; Hamdani et al., 2013; Pandit et al., 2014). In contrast, Pandit et al. and others have demonstrated that ovariectomy aggravated myofilaments Ca^2+^ sensitivity which slows down myocardial relaxation and exacerbates HFpEF (Fares et al., 2013; Pandit et al., 2014).

Taken together, the obvious cardioprotective roles of E2 exerted through the adaptive modulation of calcium handling proteins fortifies premenopausal women against the initiation and progression of HFpEF while E2 deficiency and loss of its residual protection during the postmenopausal period scaffolds a derangement in calcium homeostasis in cardiomyocytes; hence the higher incidence of HFpEF in postmenopausal women.

#### Alterations in Cardiac Cytoskeletal Titin

The cardiomyocyte cytoskeleton consists of microtubules, cytoplasmic actin, intermediate filaments, and titin. However, besides titin which provides myocardial elasticity and facilitates contractile functions, the prior mentioned cardiomyocyte cytoskeleton constituents are non-contractile (Sequeira et al., 2014). The dysregulation in myocardial contractility has been hallmarked as an underlying factor contributing to LVDD and HFpEF (Rao and Burkhoff, 2021). As such, the maladaptive alteration in titin (the contractile cytoskeleton) will be centered on, rather than the entire cardiomyocyte cytoskeleton. Titin is a giant cytoskeletal protein that plays a significant role in the elastic force of the sarcomere. The elasticity properties of titin are embedded in the I-band region, which is made up of three segments; tandem immunoglobulin (Ig) repeats, unique N2B element, and the PEVK region (LeWinter and Granzier, 2010). Titin primarily exists in two isoforms, N2B isoform (stiffer isoform) and N2BA (compliance isoform), and modulates myocardial passive stiffness either by switching isoform posttranslational modification, as well as via oxidative regulation (Linke and Hamdani, 2014). Titin can be directly phosphorylated at N2B by PKA and/or PKG as well as indirectly phosphorylated at both N2B and PEVK via βARs/PKA/CaMK in a calcium-dependent manner, besides ERK and PKCα phosphorylations (Krüger et al., 2009; Raskin et al., 2012; Hamdani et al., 2013; Hidalgo et al., 2013).

In HFpEF, reduced phosphorylation of titin by both PKA and PKG has been described as the leading cause of the increase in passive stiffness (Borbely et al., 2009; LeWinter and Granzier, 2010; van Heerebeek et al., 2012). Primarily, the dysregulation of βARs due to hyperstimulation might account for the hypophosphorylations of the N2B and PEVK spring elements of titin. βARs signaling disruption impedes the direct phosphorylation of N2B by PKA, thereby, increases the stiffness in the cardiomyocyte cytoskeleton. Also, with the signaling of βARs/PKA, which regulates intracellular Ca^2+^ being hampered, CaMK is unable to phosphorylate neither N2B nor PEVK region (Borbely et al., 2005; van Heerebeek et al., 2012). Hence, the disruption of both direct and indirect regulation of the titin aggravates myocardial stiffness, as seen in HFpEF patients. Interestingly, E2 can mitigate the dysregulation of βARs and also signal via GPR30/G_αs_/PKA (Machuki et al., 2018; Ndzie Noah et al., 2021). Thus, these cascades suggest that E2 might mask and/or complement for induction of PKA, PKC, and CaMK to phosphorylate the N2B and PEVK elements. In addition, E2, by upregulating NO, could further induce the phosphorylation of N2B via PKG (Krüger et al., 2009; Ndzie Noah et al., 2021). By these multiple mechanisms facilitated by E2, the passive stiffness of titin is normalized, thereby enhancing its physiological functions or delaying the progression of pathological stiffening of the myocardial. However, the deficiency of E2 and inhibition of ERs might abolish these bypasses to sustain the titin functions. This is further proved by relating Michalson et al. and Zile et al.’s findings. It could be inferred from their works that the deficiency of E2 and impediment in ERs signaling might contribute to the maladaptive alteration in cardiomyocyte titin (Zile et al., 2015; Michalson et al., 2018).

### Extra-Cardiomyocyte Maladaptive Alterations

#### Disruption of Extracellular Matrix Homeostasis Functions

The healthy cardiac ECM consists of non-structural and structural matricellular proteins, which are kept in a homeostatic balance by MMPs and TIMPs to preserve the myocardial architecture and its functions (Figure 1). In-depth discussions on the roles of MMPs and TIMPs have been reviewed elsewhere (Ma et al., 2014; Arpino et al., 2015; Wang and Khalil, 2018; Raeeszadeh-Sarmazdeh et al., 2020). Notably, patients presenting HFpEF are found to have stiffer myocardia resulting from excessive collagen deposition. Elucidation of the pathomechanisms resulting in the LVDD mainly implicates the dysregulation and aggravation of fibroblast activation.

Intriguingly, βARs and ERs are well expressed in fibroblasts; hence, circulating catecholamine and E2 can modulate their proliferation, differentiation to myofibroblast, and migration. As such, a typical dysregulation of the βARs in fibroblast by chronic sustained stimulation induces cascades that contribute to the adverse ECM remodeling, as reported (Jaffré et al., 2012; Lv et al., 2016; Tanner et al., 2020). Also, the deficiency of E2 and inhibition of ERs have been largely demonstrated to permit series of maladaptive cascades aggravating ECM remodeling, LV stiffness, and HFpEF. Michalson et al. in their recent study, showed that ovariectomy induced LV diastolic dysfunction in cynomolgus monkeys by increasing ECM deposition via upregulating genes encoding ECM components (COL1A2, MFAP5, and MGP) and ECM deposition promoters (CXCL12, THBS1, TIMP1, S100A4, and RPS29) (Michalson et al., 2018). Together with others, they found that during ovariectomy, synthetic myofibroblasts upregulate the synthesis of fibrillar collagen, ECM, and TIMPs while downregulating the degradative MMPs, thereby causing an imbalanced ECM turnover and exacerbating fibrosis (Dubey et al., 1998; Michalson et al., 2018). Also, cardiac collagen I and III genes were shown to increase due to E2 deficiency (Spinale et al., 1999; Dworatzek et al., 2019). Consistent with the findings of Michalson et al., El Hajj et al. also found that by antagonizing ERs, the exacerbation of cardiac structure and function were hastened in female rats (El Hajj et al., 2017). Furthermore, E2 deficiency and ERs inhibition have been shown to increase fibroblast activation and ECM depositions by; increasing NF-kB pathway activity, upregulating proinflammatory cytokine (TNFα and IL-6) secretions, and reducing NO bioavailability (McLarty et al., 2013; Vettel et al., 2014; Ambhore et al., 2019).

Within all the aforementioned studies demonstrating the adverse remodeling of the ECM due to E2 deficiency or ERs inhibition, E2 replacement and/or normalizations of ERs expression reverted all the maladaptive cascades, facilitated all the homeostatic functions that sustain the ECM (including mitigating βARs dysregulation) and improved myocardial contraction to attenuate HFpEF. Altogether, it has been suggested and shown that E2 by signaling via non-genomic (GPR30) and/or genomic (ERα and ERβ) could directly or indirectly exert these cardioprotective effects to prevent HFpEF by preserving the ECM and cardiovascular function (Watanabe et al., 2003; Wang et al., 2015; El Hajj et al., 2017). Hence, E2 deficiency in postmenopausal women increases the incidence of HFpEF by scaffolding the disruptions in homeostasis functions sustaining cardiac ECM, thereby aggravating LV stiffness and LVDD.

#### Coronary Microvascular Endothelial Dysfunctionalities

Physiological functions of coronary vessels (blood circulation in the myocardia) are dependent on functionalities of their intima layer made of endothelial cells, which in turn depend on NO bioavailability. The production of NO by eNOS is crucial for the regulation of several cardiovascular physiology such as; endothelial cell proliferation and migration, ECM degradation, angiogenesis, platelet function, vascular inflammation modulation, and vascular tone regulation (Naseem, 2005; Kolluru et al., 2010; Tousoulis et al., 2012; Ndzie Noah et al., 2021). Again, βARs and ERs are prevalent on endothelial cells; hence E2 and catecholamines are capable of indirectly regulating coronary microvessels through the facilitation of their endothelial functions (Ahmad et al., 1990; Michalson et al., 2018). In evidence to these, E2 via GPR30 was shown to activate eNOS by inducing PI3K/Phosphokinase B–mediated phosphorylation of serine 1177 (Deschamps and Murphy, 2009). Also, ERα and ERβ were shown to upregulate transcription activities that enhance endothelial nitric oxide synthase production by binding to estrogen response elements (Michalson et al., 2018). In addition, β_2_AR has been demonstrated to induce NO production via Gai/PI3K/Akt (Hu et al., 2020). Nonetheless, during chronic catecholamine stress, β_2_AR desensitization and dysregulation disrupt eNOS activation; hence, this affects the NO bioavailability, thereby causing CME dysfunction.

Disruption of NO-cGMP-PKG signaling in coronary endothelial cells delays the onset of myocardial relaxation and increases diastolic LV stiffness (Paulus, 2001; Brutsaert, 2003; LeWinter and Granzier, 2010; Paulus and Tschöpe, 2013). Therefore, the chronic inhibition of eNOS due to chronic hyperstimulation of β_2_AR might induce LVDD, as Matsubara et al. demonstrated in their study (Matsubara et al., 1998). However, it is suggested that E2 and ERs may directly enhance eNOS-NO production and as well mitigate the extent of β_2_AR dysfunctionalities to ensure the bioavailability of NO, which might help in the attenuation of LVDD/HFpEF (Huang et al., 2000; Westermann et al., 2009; Barton and Meyer, 2020; Ndzie Noah et al., 2021).

Recently, efforts to elucidate the underlying mechanisms of HFpEF are focused on coronary microvascular inflammation, besides LV afterload excess (Paulus and Tschöpe, 2013; Franssen et al., 2016; Sickinghe et al., 2019). Under proinflammatory conditions, CME cells are observed to produce reactive oxygen species (ROS), which affects NO bioavailability. This affects both endothelial cells and adjacent cardiomyocytes’ NO-cGMP-PKG signaling; hence, it delays myocardial relaxation, induces LV hypertrophy, the proliferation of fibroblasts and myofibroblasts, and also increases diastolic LV stiffness (Calderone et al., 1998; Paulus and Tschöpe, 2013; Vettel et al., 2014). On the other hand, HFpEF-induced systemic inflammation has been shown to cause CME dysfunction by increasing vascular cell adhesion molecule and E-selectin expressions. Their expression is characteristic of decreased eNOS activity and NO bioavailability. This encourages the recruitment and trafficking of circulating leukocytes to the subendothelial, which initiates the formation of atherosclerotic lesions formation and the progression of atherosclerosis which hastens the incidence of HFpEF (Venugopal et al., 2002; Kolluru et al., 2010; Westermann et al., 2011; Rush et al., 2021). These aforementioned CME dysfunctionalities have been associated with E2 deficiency. Lack of physiological levels of E2 permitted renin-angiotensin-aldosterone system-induced upregulation of ROS in cardiac and endothelial mitochondria (Alvarez et al., 2002; Rattanasopa et al., 2015). Besides, aging (menopause in women) is also shown to promote endothelial ROS production, thereby causing endothelial mitochondrial dysfunction (Rajapakse et al., 2011; Paulus and Tschöpe, 2013). Consistent with the adaptive roles of E2, its replacement inhibited renin-angiotensin-aldosterone system and ROS production, enhanced coronary blood flow as well as attenuated leukocyte activations via enhancing eNOS activity and NO bioavailability (Lang et al., 1997; Alvarez et al., 2002; Kim et al., 2006; Sabbatini and Kararigas, 2020).

Besides promoting CME eNOS-NO activities, E2 regulates vascular angiotensin II receptor type 1. The deficiency of E2 results in the upregulation of angiotensin II receptor type 1, which increases oxidative stress and disrupts CME function. Similarly, E2 replacement attenuated the oxidative stress and protected the endothelial cells from its induced apoptosis by signaling via GPR30/Akt (Nickenig et al., 1998; Sudoh et al., 2001; Xue et al., 2014). Also, E2/GPR30 has been shown to facilitate the proper formation of endothelial cell tubes (Zhou et al., 2017); this indirectly ensures coronary vascular functions are sustained.

Overall, E2 and ERs, through these mechanisms, ensure proper CME functions and attenuate the HFpEF development; however, the absence of its physiological levels in postmenopausal women predisposes them to CME dysfunctions and expedites the incidence of HFpEF.

#### Maladaptive Myocardial Inflammatory Responses

Immune cells being constituents of the myocardia and capable of crosstalk with endothelial cells, fibroblasts, and cardiomyocytes, have been extensive implicated in the exacerbation of cardiovascular diseases (Paulus and Tschöpe, 2013; Franssen et al., 2016; Adzika et al., 2019). Just as the other cellular constituents of the myocardia, cardiac resident immune cells also have profound expressions of β_2_AR and ERs. Primarily, cardiac resident immune cells have been shown to elicit hyperactive proinflammatory responses, which aggravate fibrosis and LV stiffness and increases the incidence of HFpEF via multiple mechanisms (McLarty et al., 2013; Sandstedt et al., 2019; Tanner et al., 2020). The excessive secretion of proinflammatory cytokines, IL-6 and TNFα, and the activation of NFκB (a prototypic inflammatory pathway) by immune cells induce myofibroblast differentiation, fibroblast proliferation, and migration (Sandstedt et al., 2019). While β_2_AR is implicated in the mediation of signaling in cardiomyocytes and fibroblasts during the pathological remodeling of the heart (Paur et al., 2012; Adzika et al., 2019), Tanner et al.’s recent study demonstrated that the β_2_AR upregulation of fibroblast proliferation is dependent on IL-6 secretion (Tanner et al., 2020). Hence, besides dysregulated β_2_AR, the hypersecretions of IL-6 in the myocardia have the potential of inducing excessive interstitial deposition of collagen, which distorts the myocardial architecture and causes LVDD. Furthermore, TNFα upregulation also activates cardiac fibroblast cascades that contribute to the adverse remodeling of the ECM (McLarty et al., 2013). Nonetheless, E2 and ERs exert their cardioprotective effects by inhibiting the TNFα secretions from inflammatory cells, thereby preventing fibrosis (Chancey et al., 2005; McLarty et al., 2013). Notably, NFκB regulates the expression of IL-6 and TNFα; as such, by repressing NFκB transcriptional activities, E2 and ERs can adaptively modulate inflammatory responses in the myocardia (Galien and Garcia, 1997; Arenas et al., 2005; Michalson et al., 2018; Ambhore et al., 2019). In fact, E2 and ERs have been shown to repress several inflammatory cascade pathways in the myocardia (Sabbatini and Kararigas, 2020). For example, (1) HFpEF patients are found to have elevated levels of monocyte chemoattractant protein 1, which scaffolds infiltration and accumulation of macrophage in the myocardium to promote fibrosis (Kuwahara et al., 2004). Nevertheless, E2 treatment in animal models after ovariectomy-induced LVDD was observed to downregulate sera monocyte chemoattractant protein 1 levels (Sophonsritsuk et al., 2013; Michalson et al., 2018). This implied that E2 is a potent adaptive regulator of cardiac and systemic inflammation to prevent cardiac dysfunction. (2) In the coronary vessels, E2 enhances NO bioavailability. Besides vascular tone regulation, NO exerts anti-inflammatory effects on the vascular wall, thereby attenuates pathological inflammation by preventing leukocyte interaction and transendothelial migration (Kubes et al., 1991; Venugopal et al., 2002; Westermann et al., 2009; Kolluru et al., 2010). As such, E2 indirectly attenuates HFpEF by preventing CME inflammation (Chakrabarti and Davidge, 2012). (3) Also, E2 increases angiotensin Ang 1-7 synthesis, and similar to NO, induces anti-inflammatory and antioxidative effects (Mompeón et al., 2016). (4) Lastly, adipose tissues harbor a significant amount of inflammatory cells and are a major source of inflammatory responses (Ouchi et al., 2011; Renovato-Martins et al., 2017; Srikakulapu and McNamara, 2020). Meanwhile, adipose accumulation in ovariectomy animal models and postmenopausal women is observed to increase (Lee et al., 2015; Kozakowski et al., 2017; Sharma et al., 2017). This suggests that epicardial adipose may increase during E2 deficiency and serve as a source from which inflammatory cells infiltrate the myocardia and exacerbate adverse cardiac remodeling. Consistent with the prior discussion, Mori et al. in the study demonstrated that in ovariectomized rats, cardiac inflammation and fibrosis are enhanced, resulting in diastolic dysfunction just as observed in postmenopausal women (Mori et al., 2011). However, overall, E2 treatments attenuating HFpEF, as demonstrated (Michalson et al., 2018), might involve E2-initiated inhibition of maladaptive inflammatory responses in the myocardial (including coronary vessels).

## Conclusions and Perspectives on E2 Replacement Therapy in the Management of Heart Failure With Preserved Ejection Fraction

### Heart Failure With Preserved Ejection Fraction Pathomechanism Hastened by E2 Deficiency in Brief

**FIGURE 3:**
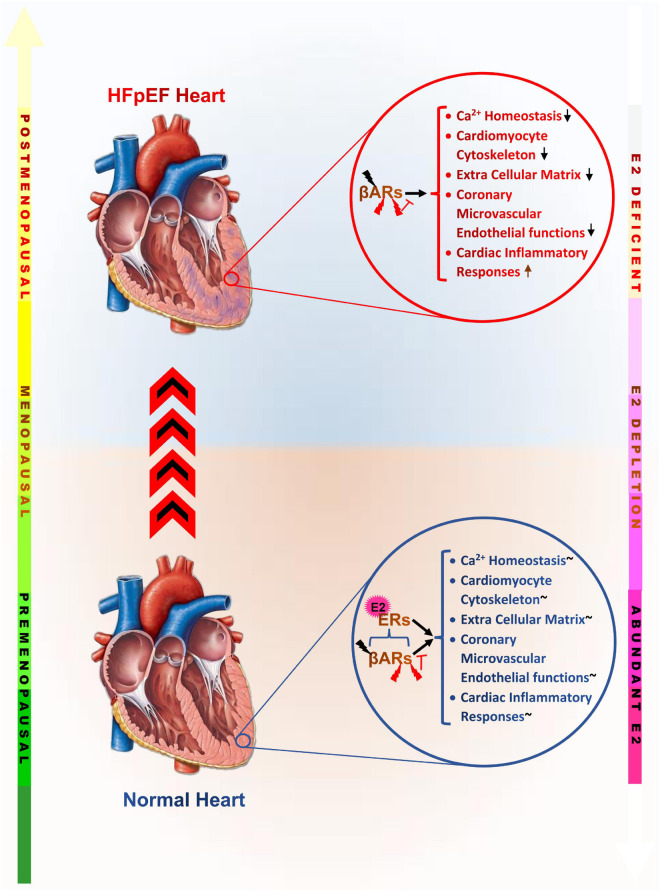
Graphical abstract of the underlying maladaptive alterations scaffolded by estrogen deficiency, which increase the incidence of heart failure with preserved ejection fraction in postmenopausal women. βARs, β-adrenergic receptors; ERs, Estrogen receptors; 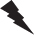, Physiological levels of catecholamine; 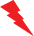, Pathological levels catecholamine; 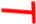, inhibition; 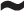, Adaptively modulated; E2, Estrogen; 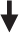, Disrupted/dysregulated; 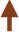, Upregulated maladaptively.

In summary, elucidating the underlying cause of the prevalence of HFpEF in postmenopausal women over the recent decades has revealed that the loss of E2 and its residual cardioprotective effects permits the initiation of maladaptive alterations intra- and extra-cardiomyocytes. These adverse alterations tend to affect the physiological functions of the cardiomyocytes as well as other constituents of the myocardia, such as; fibroblasts, inflammatory cells, coronary vessels, and epicardial adipose (Table 1). The synergy of maladaptive functions scaffolded by E2 deficiencies and ERs inactivity expedite the initiation of LVDD and progression in HFpEF as predominantly manifested in postmenopausal women (Figure 3).

**TABLE 1:** Overview of HFpEF underlying pathomechanisms scaffolded by estrogen deficiency and the ameliorative effects of estrogen therapy.

**Pathomechanisms scaffolded by estrogen deficiency**	**Cardiac health implications**	**Effects of estrogen replacement/treatment**	**Studies subject/Cells source**
βARs desensitization/downregulation during stress	βARs downstream cascades dysregulation. Impediment of cardiac inotropic and chronotropy functions. LVDD/HFpEF	Prevents βARs dysregulation. Ameliorates cardiac functions. Ameliorates LVDD/HFpEF.	Mice (Fares et al., 2013), Rats (Hou et al., 2018; Kilfoil et al., 2020), Humans (Sherwood et al., 2010).
Ca^2+^ handling dysregulation	Increased myofilaments Ca^2+^ sensitive. Distortion in myocardial contractility. Delayed myocardial relaxation. LVDD/HFpEF	Decreases myofilaments Ca^2+^ sensitive. Enhances myocardial contractility and relaxation. Ameliorates LVDD/HFpEF.	Mice (Fares et al., 2013; Parks et al., 2017), Rats (Pandit et al., 2014), Guinea pig (Yang et al., 2017), Rabbit (Matsuba et al., 2009), Monkeys (Michalson et al., 2018).
Titin dysfunction	Aggravated myocardial stiffness. LVDD/HFpEF.	Normalizes passive stiffness of titin and ameliorates LVDD/HFpEF.	Dogs (Hamdani et al., 2013), Humans (Zile et al., 2015).
Extracellular matrix distortion	Increased interstitial fibrosis. Increased myocardial stiffness. LVDD/HFpEF.	Inhibits fibrosis and preserves myocardial by regulating ECM components and deposition promoters. Ameliorates LVDD/HFpEF.	Rats (Dworatzek et al., 2019), Monkeys (Michalson et al., 2018), Humans (Zile et al., 2015; Ambhore et al., 2019).
Coronary microvascular endothelial dysfunction	Delayed myocardial relaxation. Diastolic LV stiffness. Myocardial inflammation. LVDD/HFpEF.	Enhances adaptive coronary vascular and myocardial functions. Ameliorates LVDD/HFpEF.	Rats (Alvarez et al., 2002), Monkeys (Michalson et al., 2018), Sheep (Lang et al., 1997), Humans (Alvarez et al., 2002; Mompeón et al., 2016).
Maladaptive myocardial inflammation	Aggravated fibrosis and myocardial stiffness. LVDD/HFpEF.	Modulates adaptive myocardial inflammatory responses. Attenuates fibrosis and meliorates LVDD/HFpEF.	Rats (Chancey et al., 2005; McLarty et al., 2013), Monkeys (Sophonsritsuk et al., 2013; Michalson et al., 2018), Human. (Mompeón et al., 2016).

### Prospects on Therapeutic Interventions for Heart Failure With Preserved Ejection Fraction Management

Taken together, decades of research works focused on elucidating the underlying cause of increased HFpEF in postmenopausal women have found that E2 exerts cardioprotection via the adaptive modulation of multiple homeostatic functions that sustains proper cardiac health and functions. Therefore, its deficiency during menopause permits dysregulations of cascades and physiological functions crucial for facilitating cardiac inotropy and chronotropic, should there be any stressful event.

Therapeutic interventions for the prevention and management of HFpEF in postmenopausal women has solely been the use of menopausal hormonal treatment until the findings of the Women’s Health Initiative randomized controlled trial and others, reporting the side effects and discouraged their usage significantly (Rossouw et al., 2002; Modena et al., 2005; Schierbeck et al., 2012; Antoine et al., 2016; Iorga et al., 2017; Kohn et al., 2019). Exploration of the mechanisms causing the reported side effects revealed that the late initiation of menopausal hormonal treatment and the usage of the combination of conjugated equine estrogen with medroxyprogesterone acetate as the probable causes (LaCroix et al., 2011; Maria Grazia, 2016; Hickey et al., 2018; Shufelt et al., 2018).

Also, due to the complexity of the disease mechanism of HFpEF, it is suggested that multiple pathomechanisms may be targeted with their appropriate drug combinations to improve treatment outcomes. Meanwhile, employing E2 replacement therapy might circumvent the use of multiple drugs as it has been shown to adaptively modulate these multiple pathways to exert cardioprotection and/or ameliorate adverse cardiac remodeling (Michalson et al., 2018; Sabbatini and Kararigas, 2020; Ndzie Noah et al., 2021).

Clinical data have shown that the severity of LVDD/HFpEF is increased mostly after 6 years of menopause (Gerber et al., 2015; Pfeffer et al., 2019). In line with these reports, it has been shown in ovariectomized monkeys that when E2 replacement therapies were initiated late (4.5 years) after ovariectomy, E2 was ineffective at attenuating maladaptive inflammatory responses, disruption of ECM, and dysregulation of Ca^2+^ handling. Conversely, when the E2 replacement therapy was initiated early (1 month) after ovariectomy, E2 was potent at inhibiting the aforementioned dysfunctions, which hasten the incidence of HFpEF (Sophonsritsuk et al., 2013; Michalson et al., 2018). These studies have further suggested that E2 replacement therapy initiated early (within 5-6 years of menopause) has more efficacy in improving heart function and preventing LVDD/HFpEF while circumventing any treatment side effects (Shao et al., 2012; Sophonsritsuk et al., 2013; Hodis et al., 2016; Michalson et al., 2018). Therefore, E2RT might not be effective for treating the latter stages of HFpEF. Therefore, it is proposed that E2 replacement therapy targeted at the attenuation of HFpEF in postmenopausal women should be initiated within the critical window of 5-6 years after menopause.

## Author Contributions

AOA conceived the review idea. GKA and AOA fine-tuned the review idea and drafted and wrote the manuscript. GKA, AOA, RM, MLNN, JA-A, RR, and NA proofread and revised the manuscript with the supervision of HS. All authors accepted the final version of the manuscript.

## Conflict of Interest

The authors declare that the research was conducted in the absence of any commercial or financial relationships that could be construed as a potential conflict of interest.

## Publisher’s Note

All claims expressed in this article are solely those of the authors and do not necessarily represent those of their affiliated organizations, or those of the publisher, the editors and the reviewers. Any product that may be evaluated in this article, or claim that may be made by its manufacturer, is not guaranteed or endorsed by the publisher.
